# Phenotypic and Molecular Detection of Biofilm Formation in *Staphylococcus aureus* Isolated from Different Sources in Algeria

**DOI:** 10.3390/pathogens9020153

**Published:** 2020-02-24

**Authors:** Rachid Achek, Helmut Hotzel, Ibrahim Nabi, Souad Kechida, Djamila Mami, Nassima Didouh, Herbert Tomaso, Heinrich Neubauer, Ralf Ehricht, Stefan Monecke, Hosny El-Adawy

**Affiliations:** 1 Faculty of Nature and Life and Earth Sciences, Djilali-Bounaama University, Soufay, Khemis-Miliana 44225, Algeria; nassimadidouh@gmail.com; 2 Institute of Bacterial Infections and Zoonoses, Friedrich-Loeffler-Institut, 07743 Jena, Germany; helmut.hotzel@fli.de (H.H.); herbert.tomaso@fli.de (H.T.); heinrich.neubauer@fli.de (H.N.); 3 Faculty of Sciences, Yahia-Farès University, Urban Pole, 26000 Médéa, Algeria; ibrahiim.nabi@gmail.com (I.N.); kechiida.souaad@gmail.com (S.K.); djamilamami23@gmail.com (D.M.); 4 Leibniz Institute of Photonic Technology (IPHT), 07745 Jena, Germany; ralf.ehricht@leibniz-ipht.de (R.E.); stefan.monecke@leibniz-ipht.de (S.M.); 5 InfectoGnostics Research Campus Jena e. V., 07743 Jena, Germany; 6 Institute for Physical Chemistry, Friedrich-Schiller-University, 07743 Jena, Germany; 7 Faculty of Veterinary Medicine, Kafrelsheik University, Kafr El-Sheik 35516, Egypt

**Keywords:** *Staphylococcus aureus*, biofilm formation, adhesion genes, microarray assay, Algeria

## Abstract

*Staphylococcus aureus* is an opportunistic bacterium causing a wide variety of diseases. Biofilm formation of *Staphylococcus aureus* is of primary public and animal health concern. The purposes of the present study were to investigate the ability of *Staphylococcus aureus* isolated from animals, humans, and food samples to form biofilms and to screen for the presence of biofilm-associated and regulatory genes. In total, 55 *Staphylococcus aureus* isolated from sheep mastitis cases (*n* = 28), humans (*n* = 19), and from food matrices (*n* = 8) were identified using matrix-assisted laser desorption/ionization time-of-flight mass spectrometry (MALDI-TOF MS). The ability of *Staphylococcus aureus* for slime production and biofilm formation was determined quantitatively. A DNA microarray examination was performed to detect adhesion genes (*ica*ACD and biofilm-associated protein gene (*bap*)), genes encoding microbial surface components recognizing adhesive matrix molecules (MSCRAMMs), regulatory genes (accessory gene regulator (*agr*) and staphylococcal accessory regulator (*sar*A)), and the staphylococcal cassette chromosome *mec* elements (SCC*mec*). Out of 55 *Staphylococcus aureus* isolates, 39 (71.0%) and 23 (41.8%) were producing slime and biofilm, respectively. All *Staphylococcus aureus* strains isolated from food showed biofilm formation ability. 52.6% of the *Staphylococcus aureus* strains isolated from sheep with mastitis, and 17.9% of isolates from humans, were able to form a biofilm. Microarray analysis typed the *Staphylococcus aureus* into 15 clonal complexes. Among all *Staphylococcus aureus* isolates, four of the human isolates (21.1%) harbored the *mec*A gene (SCC*mec* type IV) typed into 2 clonal complexes (CC22-MRSA-IV and CC80-MRSA-IV) and were considered as methicillin-resistant, while two of them were slime-producing. None of the isolates from sheep with mastitis harbored the *cna* gene which is associated with biofilm production. The *fnb*B gene was found in 100%, 60% and 40% of biofilm-producing *Staphylococcus aureus* isolated from food, humans, and sheep with mastitis, respectively. Three *agr* groups were present and *agr* group III was predominant with 43.6%, followed by *agr* group I (38.2%), and *agr* group II (18.2%). This study revealed the capacity of *Staphylococcus aureus* isolates to form biofilms and highlighted the genetic background displayed by *Staphylococcus aureus* isolates from different sources in Algeria.

## 1. Introduction

*Staphylococcus* (*S*.) *aureus* is a bacterium that can cause a wide range of diseases in humans and animals. The capacity of *S. aureus* to colonize and infect different sites of the body is related to the presence of a variety of virulence factors [1]. Among the virulence factors responsible for the pathogenicity of *S. aureus*, adhesion proteins and the ability to form biofilms on biotic and abiotic surfaces are of importance [2,3]. In human medicine, large numbers of infections are associated with biofilms produced by staphylococci. The spectrum of clinical presentation comprises osteomyelitis, endocarditis, and other medical device-related infections such as prosthetic joints, endotracheal tubes, skeletal prostheses, vascular catheters, cardiac pacemakers, and heart valves [4,5]. Biofilms formed by *S. aureus* on food-processing surfaces constitute a critical issue for the food industry and is a dangerous source of contamination of food matrices and human handlers [6]. *Staphylococcus aureus* is one of the most frequent causes of mastitis in dairy cattle and sheep [7,8,9]. The severity of the disease and the clinical presentation depends on the production of several toxins and adhesion proteins [10].

Two aspects of biofilm play major roles in the pathogenesis of human and animal infections: first, the adhesion of the bacteria to epithelial cells thus facilitates the insertion and the release of different toxins [4,11,12]. Second, decreased diffusion of antimicrobial molecules into the biofilm matrix limits the effectiveness of antibiotic therapy [13,14]. Moreover, in the food industry, biofilms on surfaces increase the resistance to disinfectant agents [15].

A biofilm was defined as a structured community of microbial cells embedded in a self-produced matrix of extracellular polymeric substances, which is attached to a surface [16]. The biofilm-building process is divided into distinct phases beginning with attachment of staphylococcal cells on a biomaterial, followed by accumulation and biofilm maturation. The dispersal stage corresponds to the dissemination of bacterial cells that contaminate other surfaces [15,17]. In staphylococcal biofilms, the attachment to biotic or abiotic surfaces is facilitated via microbial surface components that recognize adhesive matrix molecules (MSCRAMMs) and the biofilm-associated protein (Bap) [18]. The accumulation phase appears to be dependent on polysaccharide intercellular adhesin (PIA) encoded by the *ica*ADBC operon [17].

The formation of biofilms is a complex process depending on several factors like nutrient availability, pH value, oxygen level, and surface properties [18]. In addition, biofilm formation in *S. aureus* is under the control of two genetic loci, *sar*A (staphylococcal accessory regulator) and *agr* (accessory gene regulator) quorum-sensing system [19,20].

In Algeria, pathogenicity and antimicrobial resistance of staphylococci were investigated in clinical and food samples [21,22,23]. However, the biofilm-forming ability of *Staphylococcus* isolates and their genetic determinants were rarely evaluated in Algeria [24]. The aim of the present study was to investigate the phenotypic biofilm formation of *S. aureus* isolated from different sample types and to assess the presence of genes that are typically associated with biofilm formation. 

## 2. Materials and Methods 

### 2.1. Bacterial Isolates 

Fifty-five *S. aureus* isolates were chosen from a previous screening study in Algeria [9,21] to assess their biofilm production ability. The bacterial strains were isolated from food matrices (*n* = 8), humans (*n* = 19; 3 with clinical infections and 16 from nasal carriage), and from cases of mastitis in sheep (*n* = 28). Briefly, a total of 112 food samples were taken from several commercial points and local districts located in Médéa and Ain-defla regions in northern Algeria. Samples were collected from different matrices. For solid food matrices (minced beef meat, sausages, chicken meat, and creamery cake) and liquid food matrix (milk), quantities of 100 g and 100 ml were collected in sterile containers and transferred at 4 °C to the laboratory for microbiological examination. The food samples were processed according to ISO 6888-1/A1 [25] using Baird-Parker medium supplemented with 5% of egg yolk emulsion (Oxoid Ltd., Basingstoke, UK) and 0.5% potassium tellurite.

In total, 71 nasal swabs were collected from volunteer persons who had contact with livestock animals (veterinarians and farmers) to screen the staphylococci nasal carriage. Different clinical samples (wounds, vaginal discharge, and catheter tips) were collected from hospitalized patients in the Médéa and Ain-defla regions. The nasal samples were obtained with a sterile cotton swab and the clinical samples were processed in the same day in the laboratory for microbiology.

In this study, 123 milk samples were aseptically collected from 110 sheep farms in the two provinces of Médéa and Ain-Defla in northern Algeria. Milk samples were collected from sheep presenting clinical symptoms of acute mastitis (fever, edema, anorexia with flock abandonment or suckling refuse, udder pain, consistency or discoloration of udder and bloody secretion). Milk samples were transported in ice-cooled containers to the microbiology laboratory. 

The bacteriological analyses of samples from humans and sheep mastitis cases (nasal swabs, clinical samples, and milk) were performed according to conventional methods, as described previously [26,27]. Specimens were transferred to Brain Heart Infusion (BHI) broth and incubated overnight at 37 °C. One hundred μl of broth culture were streaked out on Columbia agar (Oxoid Ltd., Basingstoke, UK), supplemented with 5% sheep blood, and incubated at 37 °C for 24 to 48 h. Subsequently, the colonies were picked and subcultured on Mannitol Salt Agar (MSA) (Oxoid Ltd., Basingstoke, UK). Single colonies on MSA agar were subjected to Gram staining, catalase, and coagulase tests according to standard microbiological methods [28].

### 2.2. Ethics Statement

Study protocol was approved by Medical Ethics Research Committee of the Yahia Farès University, Urban Pole, Médéa, Algeria, and from the managers of the hospital in which the study was conducted. Informed written consent was obtained from each participant in the study. Confidentiality and personal privacy was respected in all levels of the study. Collected data will not be used for any other purpose.

### 2.3. Matrix-Assisted Laser Desorption/Ionization Time-of-Flight Mass Spectrometry (MALDI-TOF MS) Identification

The identification of bacterial species was performed by matrix-assisted laser desorption/ionization time-of-flight mass spectrometry (MALDI-TOF MS) according to the procedure described previously [29]. Briefly, a water suspension of cultured bacteria (300 μl) was precipitated with 900 µL of ethanol (96% vol/vol; Carl Roth GmbH, Karlsruhe, Germany) and centrifuged for 5 min at 10,000 × g. The pellet was resuspended in 50 μl of 70% (vol/vol) formic acid (Sigma-Aldrich Chemie GmbH, Steinheim, Germany), followed by adding 50 μl of acetonitrile (Carl Roth GmbH), mixing, and centrifugation for 5 min at 10,000 × g. Then, 1.5 μl of the supernatant were spotted onto a MTP 384 Target Plate Polished Steel TF (Bruker Daltonik GmbH, Bremen, Germany) and air-dried. The dried material was overlaid with 2 μl of a saturated solution of α-cyano-4-hydroxycinnamic acid (in a solution of 50% acetonitrile, 2.5% trifluoroacetic acid) (Sigma-Aldrich Chemie GmbH, Steinheim, Germany). Air-drying at room temperature followed. Spectra recording and data analysis were done with an Ultraflex instrument and Biotyper 3.1 software (Bruker Daltonik GmbH) [30].

### 2.4. Phenotypic Detection of Slime Production Ability on Congo Red Agar

For testing the ability of slime production, the Congo Red Agar (CRA) assay was used as previously described [31]. The isolates were inoculated on CRA plates (BHI agar supplemented with 0.8 g/L Congo Red and 50 g/L of saccharose) and incubated at 37 °C for 24 h. The phenotypic identification of slime-producing colonies was performed according to Arciola et al. [32], where slime-producing isolates form black colonies and non-producing strains develop red colonies. Colonies of variable phenotypes (e.g., red colonies with a black center) were considered as slime-producing as described by Touati et al. [33].

### 2.5. Assessment of Biofilm Production by Tissue Culture Plate Assay

The ability of *S. aureus* isolates to form biofilms was investigated quantitatively by applying a Tissue Culture Plate (TCP) assay in 96-well microtiter plates as described previously [34]. In brief, isolated colonies of *S. aureus* from fresh cultures were inoculated in 5 ml of Trypticase Soy Broth (TSB) and incubated overnight at 37 °C. The cultured suspensions were diluted 1:50 with TSB-1% glucose medium and 150 µL of the suspension were filled (in triplicates) into wells of a sterile 96-U-bottom polystyrene plate (PorLab Scientific Co., Ltd, Nanjing, China). TSB-1% glucose served as a negative control. After 24 h of incubation at 37 °C, the content of the wells was gently removed, and washed with 200 μl of phosphate-buffered saline (PBS; 7 mM Na_2_HPO_4_, 3 mM NaH_2_PO_4_, and 130 mM NaCl, pH 7.4). Adherent bacteria were fixed with methanol (p.a.) and stained with 0.1% crystal violet (150 µL/well) for 15 min. Three gentle washing steps followed to remove the excess of stain and the plate was kept for heat-drying (60 °C for 60 min). The fixed crystal violet was solubilized by adding 150 µL of 96% ethanol, covered, and held without shaking for 30 min. Measurement of the optical density (OD) was performed at 590 nm using a microtiter plate reader MP96 (Safasmonaco, Monaco, France). Interpretation of biofilm production was done as described previously [34]. The optical densities of tested isolates were compared to OD of the negative control (ODc). The biofilm-producing isolates were categorized into four classes as follows: non-producers (OD < ODc), weak (ODc < OD < 2ODc), moderate (2ODc < OD < 4ODc), and strong (OD > 4ODc).

### 2.6. DNA Microarray Analysis

The detection of biofilm-associated genes (*ica*ACD and *bap*), genes encoding MSCRAMMs (*bbp*, *clf*AB, *cna*, *ebh*, *ebp*S, *eno*, *fib*, *fnb*AB, *map*, *sas*G, *sdr*CD and *vwb*), and regulatory genes (*agr*I, II, III, IV and *sar*A), as well as SCC*mec* typing were done using the *S. aureus* specific DNA microarray assay (StaphyType; Abbott (Alere Technologies GmbH), Jena, Germany) according to the procedure described previously [35]. Briefly, purified DNA (after bacterial lysis) was obtained using an automated EZ1 system (Qiagen, Hilden, Germany) according to the protocol supplied by the manufacturer. DNA samples were subjected to a linear primer elongation using one primer per target. Biotin-16-dUTP was incorporated into the amplicons. Labeled samples hybridized stringently to the probes of the microarray. After washing, horseradish-peroxidase-streptavidin conjugation and staining by precipitation of a dye were performed. The obtained image was analyzed automatically using a reader and software provided by Abbott (Alere Technologies GmbH). Data interpretation was performed as described previously [36,37]. 

### 2.7. Statistical Analysis

Statistical analysis was performed with StatView^TM^ software (SAS Institute Inc., Cary, North Carolina, USA). The differences in the degree of slime production and biofilm formation between groups were examined by Fisher’s exact test that can be done online at https://www.langsrud.com/stat/fisher.htm. Results were considered statistically significant at a *p* value of < 0.05.

## 3. Results

In total, 55 *S. aureus* isolates were recovered. Eight *S. aureus* were isolated from food (1 creamery cake, 5 raw milk, and 2 minced beef meat), 16 *S. aureus* isolates were obtained from nasal carriage, 3 from clinical infections of humans, and 28 *S. aureus* isolates were identified from sheep mastitis cases.

Twenty-three out of 55 *S. aureus* isolates (41.8%) were found to be biofilm-producing. All food isolates showed biofilm formation ability. Seven (87.5%) strains were firmly adherent, and one (12.5%) was moderately adherent. Most *S. aureus* isolates from sheep mastitis were identified as non-adherent 23 (82.1%), and only 5 (17.9%) of them were biofilm producers. Ten (52.6% ) *S. aureus* isolates from humans were adherent (7 isolates were weakly adherent, and 2 were strongly adherent) (Table 1).

In total, 39 (70.9%) of the isolated *S. aureus* were found to be slime-producing in the Congo Red test. All *S. aureus* isolated from food were slime-producing, whereas 18 (64.3%) and 13 (68.4%) of sheep and human isolates showed slime production, respectively (Table 1). No significant difference between animal and human isolates was observed in slime production (*p* > 0.05).

In a cross-analysis of slime and biofilm production ability with two assays (CRA and TCP), all food isolates were simultaneously biofilm and slime producers. Only two isolates (7.1%) from sheep mastitis and 8 (42.1%) from humans were found to be TCP-positive and slime producers.

The results of DNA microarray analysis showed that all isolates from sheep mastitis cases and food samples were identified as methicillin-sensitive *S. aureus* (MSSA), while four (21.1%) *S. aureus* strains isolated from humans were methicillin-resistant *S. aureus* (MRSA), harboring the *mec*A gene (SCC*mec* type IV). Among the four MRSA isolates (isolated from nasal swabs), two were weakly biofilm-producing with slime production, and the others neither showed biofilm production nor slime production.

Subsequent microarray analysis typed the MSSA into 15 different clonal complexes (CCs) (5 in sheep, 10 in humans, and 4 in food) (Table 2). The ruminant leukocidin genes *luk*F-P83 and *luk*M were found in 27 (25 sheep, 1 human, and 1 food) isolates belonging to CC130/521, CC479, CC522, and CC705. Two MSSA isolates (1 from sheep and 1 from human) were not typable. Four MRSA isolated from humans were typed into 2 CC (CC22-MRSA-IV and CC80-MRSA-IV). The Panton-Valentine Leukocidin (PVL) gene was detected only in one MRSA isolated from a human.

Isolates were assigned to three *agr* groups: the predominant *agr* group was *agr*III (43.6%), followed by *agr*I (38.2%), and *agr*II (18.2%). The regulatory gene *sar*A was detected in all isolates (Table 3 and Table 4).

The distributions of biofilm-associated genes, genes encoding MSCRAMMs, and regulatory genes among isolates regarding biofilm and slime formation are summarized in Table 3 and Table 4, respectively. All isolates harbored *ica*ACD genes but the *bap* gene was not found.

For MSCRAMMs genes, *clf*A/B*, ebp*S*, eno, fnb*A*, map, sdr*C*,* and *vwb* genes were detected in all isolates, regardless of their origin or ability to form biofilms. In biofilm-producing isolates originating from sheep mastitis, the *cna* gene was not detected, while the *cna* gene was found in 50% of biofilm-producing isolates from humans (5/10) and food (4/8). The *fnb*B gene was identified in all food isolates and moderately detected in 40% (2/5) in sheep mastitis biofilm-producing isolates. 

The results revealed that *agr*I, *agr*II, and *agr*III types were detected in 7 (1 moderate and 6 weak adherent), 6 (4 strong and 2 weak adherent), and 6 (4 strong and 2 moderate adherent) biofilm-producing *S. aureus* isolates, respectively.

Sixty percent (3/5) of biofilm-producing isolates from sheep mastitis cases and 20% (2/10) of isolates from humans belonged to *agr*III. In biofilm-producing *S. aureus* isolated from humans, *agr*I was predominantly detected in five isolates (50%), followed by *agr*II in 3 isolates (30%). In biofilm-producing *S. aureus* isolated from food samples, *agr*II and *agr*III were equally detected (37.5%), while *agr*I was found in 2 (25%) isolates.

The *cna, fnb*B, and *sas*G genes were occasionally detected in *S. aureus* isolated from sheep mastitis (21.4%, 17.9%, and 32.1%, respectively), but frequently among human isolates, in either slime producers or non-slime producers.

The *cna* gene was detected in 8 (61.5%), 4 (50%), and 4 (22.2%) of slime-producing *S. aureus* originating from humans, food, and sheep mastitis, respectively. The *fnb*B gene was found in 100%, 61.5%, and 16.7% of slime-producing *S. aureus* isolated from food isolates (8/8), humans (8/13), and sheep mastitis (3/18), respectively.

The *sdr*D gene was detected in 42 isolated *S. aureus* in this study. The *sdr*D gene was found in 12 (92.3%), 7 (87.5%), and 18 (72.2%) of slime-producing *S. aureus* originating from humans, food, and sheep mastitis, respectively. Noticeably, the *sdr*D gene was found more frequently in slime producers than in non-slime producers (*p* > 0.05).

The results revealed that the *agr*I, *agr*II, and *agr*III genes were detected in 14 (35.9%), 7 (17.9%), and 18 (46.2%) slime-producing *S. aureus*, respectively.

Among the slime-producing isolates assigned to *agr*III, 66.7% (12/18), 37.5% (3/8), and 23.1% (3/13) were obtained from sheep mastitis, food, and humans, respectively. In slime-producing *S. aureus* isolated from humans, *agr*I was detected in 7 isolates (53.8%) followed by *agr*II and agrIII in 3 isolates (23.1%). In slime-producing *S. aureus* isolates from food samples, *agr*II and *agr*III were equally often detected (37.5%), while *agr*I was found in 25% of the isolates. 

## 4. Discussion

*Staphylococcus aureus* is a zoonotic pathogen capable of causing a wide variety of syndromes in animals and humans. Biofilm-producing *S. aureus* is considered as a major public and animal health concern. The objectives of this study were to investigate the biofilm-forming ability of *S. aureus* isolated from different sources and to analyze biofilm-associated and regulatory genes using microarray analysis.

In this study, all isolates from food showed higher biofilm production than those from humans (52.6%) and sheep mastitis cases (17.9%). This result was in concordance with the study of Rodriguez-Lazaro et al. [38]. Most of *S. aureus* isolates from sheep mastitis did not form biofilms (82.1%), which was in concordance with a previous report on *S. aureus* isolates from sheep milk [39]. In the present study, 52.6% of human isolates were biofilm-producing *S. aureus*. Concordantly, it was reported that isolates able to produce biofilm were more often found among clinical isolates from humans than in commensal isolates [40].

The CRA assay showed that slime production occurred frequently (70.1%) and isolates from food are more likely to be slime producers (100%) than isolates from humans (68.4%) or sheep mastitis (64.3%) isolates. The present findings were in concordance with studies of Ammendolia et al. for human clinical isolates (88.9%) [41] and Duran et al. for isolates from nasal samples (58.3%) [42].

The cross-analysis of biofilm-formation and slime production ability using the results of TCP and CRA assays revealed no perfect correlation in *S. aureus* isolated from humans (52.6% versus 68.4%) and sheep mastitis cases (17.9% versus 64.3%), but matched perfectly in food. This finding corroborates the results of a previous study on *S. aureus* strains, in which isolates from bovine mastitis were more frequently slime-producing (CRA assay) than biofilm-forming isolates on TCA (91.4% versus 68.6%) [43].

Subsequent microarray analysis showed high genetic diversity among isolated *S. aureus* from different sources in this study.

In this study, *S. aureus* isolates were genetically diverse (15 CCs). The ruminant leukocidin genes *luk*F-P83 and *luk*M were found in 27 isolates (25 sheep, 1 human, and 1 food). The Panton-Valentine-Leukocidin (PVL) gene, which is mostly associated with community-acquired MRSA infections, was detected only in one MRSA isolated from a human.

Previous investigations showed that the intracellular adhesion (*ica*ACD) gene products are important in the ability of *S. aureus* biofilm formation [44,45]. The presented data of the DNA microarray analysis revealed that all *S. aureus* isolates harbored *ica*ACD genes regardless of their origin. These results were in agreement with several findings made in human infections [46], human nasal samples [47], sheep mastitis [48], and isolates from food matrices [6].

Here, not all *ica*ACD gene-carrying isolates had the capacity to produce biofilms and/or slime. It was reported that the correlation between *ica* carriage and biofilm-forming ability in *S. aureus* is unpredictable [3], because the expression of biofilm-depending genes and adhesion on surfaces is a complex process of gene regulation dependant on several factors as well as nutrients, pH value, and surface characteristics [15,18,19,49].

In this study, presence of the *bap* gene was not found in any of the isolates. In general, the *bap* gene, which promotes inert surfaces and intracellular adhesion, is rarely observed in *S. aureus* [39,50] and its presence has only been reported in few *S. aureus* isolates from cases of bovine and sheep mastitis [39,51,52].

On the other hand, few extracellular proteins as well as cell-bound MSCRAMMs are considered essential for the pathogenicity of *S. aureus*. Indeed, genes like *fnb* (A and B), *clf*A*,* and *cna* that encode MSCRAMMs are responsible for adhesion to host cells, and may participate in biofilm formation [53]. In *S. aureus, fnb*AB genes encode for fibronectin-binding proteins, which are functioning as adhesins and invasins to modulate adherence and internalization, and it has also been reported to facilitate biofilm assembly [53,54].

FnBPA and FnBPB proteins are involved in biofilm maturation but not in primary attachment. They are generally involved in the *ica*-encoded polysaccharide intercellular adhesion (PIA) [54]. This function was confirmed by a report in which a primary attached biofilm was damaged by *fnb*AB mutation [53]. The findings of this study showed that *fnb*A and *fnb*B were detected in 100% and 41.8% of all *S. aureus* isolates, respectively. In a previous study, *fnb*A and *fnb*B genes were detected in 77.8% and 81% of MRSA isolates from patients, respectively [55]. Here, the *fnb*B gene was rarely detected among non-biofilm-producing isolates but found in all biofilm-producing isolates from food matrices and humans. These findings were in discordance with a previous reports where either the *fnb*B gene was not detected in biofilm-producing isolates [6] or detected in a low level (29.0%) in MRSA isolated from humans [50]. However, a previous study reported that *fnb*B occurred frequently (99.5%) in *S. aureus* isolated from humans [46].

Clumping factors ClfA and ClfB encoded by the genes *clf*A and *clf*B are the most important proteins for the binding of *S. aureus* to fibrinogen and fibrin [56]. In this study, all *S. aureus* harbored *clf*AB genes, which is contradictory to reports on sheep mastitis isolates [7] and isolates from human nasal and clinical samples [50].

Our findings showed that 57.1% of biofilm-producing isolates from food carried the *cna* gene, while in previous studies, it was reported that 80% of the biofilm-forming isolates from food were *cna*-positive [6] and 73% of the clinical isolates [55]. Contrary to our results, another study showed that only 17% of *S. aureus* isolated from sheep mastitis possessed the *cna* gene, suggesting that the *cna* gene was detected only in some clonal complexes of *S. aureus* [57].

The *sas*G gene (surface protein of *S. aureus*) was shown to promote the formation of biofilms [58]. The present findings revealed that the *sas*G gene was frequently detected in the biofilm-producing *S. aureus* independent from the MSCRAMMs genetic performance. Indeed, the product of *sas*G has the ability to mask other binding components of *S. aureus* to microbial surface components (MSCRAMMs), including *clf*B, and limits their promoting to form biofilms [56,59]. Furthermore, *sas*G promotes strong adhesion affinity, even with masking *clf*B and independent of the *ica*-encoded PIA with its masking properties. The *sas*G biofilm formation was clearly shown to be dependent on the level of *sas*G expression [59].

In *S. aureus*, the accessory gene regulator (*agr*) and the staphylococcal accessory regulator (*sar*A) are genetic loci that determine the regulation and control of the expression of several virulence determinants [57]. Both *sar*A and *agr* play global regulatory roles in the limitation and/or propagation of *S. aureus* biofilm formation [20,60].

The biofilm-producing *S. aureus* strains isolated from food in this study carried *agr*I, II, or III, while other studies found that the majority (60%) of biofilm-producing *S. aureus* were the carriers of *agr*III [6]. In *S. aureus* isolated from humans in this study, the *agr*I was more predominant in biofilm producers followed by *agr*II, which disagreed with a previous study in Palestine that reported biofilm-producing *S. aureus* isolated from humans mainly possessed *agr*I and *agr*III [50]. It was reported that *agr* expression can restrict biofilm formation allowing the colonization of a new surface by dispersion of the bacterium from a previously formed biofilm [12,61].

The *sar*A gene is strongly related to the polysaccharide poly-N-acetylglucosamine (PNAG)- dependent biofilm formation in *S. aureus* [60,62]. In this study, all isolates carried the *sar*A gene independent of their ability to form biofilms. The detection of *sar*A is likely correlated to the detection of *ica*ACD loci, as demonstrated by previous findings [55,60,62] where isolates harboring *ica*ADBC genes were found to be positive for the *sar*A gene and isolates of the *ica*ADBC-negative genotype did not carry *sar*A. 

The biofilm formation ability of *S. aureus* isolated from sheep mastitis cases was detected in 17.9% of isolated strains consolidated the fact that pathogenesis of udder infection critically depends on adhesion of *S. aureus* to the mammary gland parenchyma. In the same tendency, the binding and/or insertion ability of *S. aureus* to mammary gland epithelium was related to the production of biofilms [13]. In addition, biofilm formation limits antimicrobial agents therapy of mastitis by reducing the diffusion of antibiotics through the biofilm matrix [14,63].

In conclusion, *S. aureus* as known pathogenic bacteria can induce gene expression of biofilm that has an important role in the pathogenesis of staphylococcal infections and causes bacterial attachment and colonization on tissues or surfaces that may act as a substrate for microbial adhesion. This study revealed the biofilm-forming ability of *S. aureus* isolated from human, animal, and food samples. It represents an extensive analysis of several potential biofilm-associated-genes in *S. aureus* isolates. The results of this study proved that MSSA isolates from food origin have significant capacity for forming biofilms, which can play a key role for the successful dissemination of MSSA lineages via food.

Therefore, significant differences in gene carriage between biofilm-forming and non-biofilm-forming isolates were not observed.

To the best of our knowledge, the current study is the first of its kind in Algeria to evaluate the biofilm and slime-forming abilities among *S. aureus* recovered from different origins.

## Figures and Tables

**Table 1:** Distribution of biofilm and slime-producing *S. aureus* isolated from sheep, humans, and food samples.

Criteria			Sheep (28) n (%)	Human (19) n (%)	Food (8) n (%)
Biofilm-producing (TCP performance) *	Positive	Strong	0	2 (10.5)	7 (87.5)
		Moderate	2 (7.14)	1 (5.3)	1 (12.5)
		Weak	3 (10.7)	7 (36.8)	0
		Total	5 (17.9)	10 (52.6)	8 (100)
	Negative		23 (82.1)	9 (47.4)	0
Slime-producing (CRA performance) **	Slime	Positive	14 (50.0)	9 (47.4)	8 (100)
		Variable	4 (14.3)	4 (21.1)	0
		Total	18 (64.3)	13 (68.4)	8 (100)
	No slime		10 (35.7)	6 (31.6)	0
Biofilm and slime-producing (TCP and CRA)			2 (7.1)	8 (42.1)	8 (100)

* TCP: Tissue Culture Plate; ** CRA: Congo Red Agar

**Table 2:** Genetic diversity of *Staphylococcus aureus* isolates.

Clonal Complex	Sheep (*n* = 28)	Human (*n* = 19)	Food (*n* = 8)	Comments
CC1-MSSA	0	2 (10.5%)	3 (37.5%)	Common lineage in cattle and in humans
CC5-MSSA	0	1 (5.3%)	2 (25.0%)	Common lineage in humans and in poultry
CC8-MSSA	1 (3.6%)	0	0	Common human lineage, no data on sheep
CC15-MSSA	0	1 (5.3%)	0	Common human lineage
CC22-MSSA	0	4 (21.1%)	0	Common human lineage
CC22-MRSA-IV (*tst* 1+)	0	3 (15.8%)	0	Common MRSA strain in humans from the Middle East and the Mediterranean
CC30-MSSA	0	1 (5.3%)	0	Common human lineage
CC45-MSSA		2 (10.5%)		Common human lineage
CC80-MRSA-IV (PVL+)	0	1 (5.3%)	0	Common MRSA strain in humans from the Middle East and the Mediterranean
CC97-MSSA	1 (3.6%)	1 (5.3%)	2 (25.0%)	Common lineage in cattle and in humans
CC130/521-MSSA	18 (64.3%)	0	0	Common lineage in sheep/goats
CC398-MSSA	0	1 (5.3%)	0	Common lineage in poultry and increasingly in humans
CC479-MSSA	2 (11.1%)	0	1 (12.5%)	Common lineage in cattle
CC522-MSSA	4 (14.3%)	0	0	Common lineage in sheep/goats
CC705-MSSA	1 (3.6%)	1 (5.3%)	0	Common lineage in cattle, unusual in humans, no sufficient data on sheep
Not identified	1 (3.6%)	1 (5.3%)	0	
Total CCs detected	5	10	4	

**Table 3:** Correlation between biofilm formation ability using TCP assay and distribution of biofilm-associated genes, microbial surface components that recognize adhesive matrix molecules (MSCRAMMs) genes, and regulatory genes in the tested *S. aureus* isolates.

Criteria			Sheep Mastitis Isolates (*n* = 28)				Human Isolates (*n* = 19)				Food Isolates (*n* = 8)			
			Absence (23)	Weak (3)	Moderate (2)	Strong (0)	Absence (9)	Weak (7)	Moderate (1)	Strong (2)	Absence (0)	Weak (0)	Moderate (1)	Strong (7)
**Polysaccharide intercellular adhesin and biofilm associated protein genes**	*ica*ACD		23 (100%)	3 (100%)	2 (100%)	0	9 (100%)	7 (100%)	1 (100%)	2 (100%)	0	0	1 (100%)	7 (100%)
	*bap*		0	0	0	0	0	0	0	0	0	0	0	0
**Genes encoding microbial surface components recognizing adhesive matrix molecules (MSCRAMMs)**	*bbp*		23 (100%)	2 (66.6%)	2 (100%)	0	8 (88.8%)	7 (100%)	1 (100%)	2 (100%)	0	0	1 (100%)	5 (71.4%)
	*clf*A/B		23 (100%)	3 (100%)	2 (100%)	0	9 (100%)	7 (100%)	1 (100%)	2 (100%)	0	0	1 (100%)	7 (100%)
	*cna*		6 (26%)	0	0	0	8 (88.8%)	3 (42.8%)	1 (100%)	1 (50%)	0	0	0	4 (57.1%)
	*ebh*		23 (100%)	3 (100%)	2 (100%)	0	5 (55.5%)	4 (57.1%)	1 (100%)	2 (100%)	0	0	1 (100%)	7 (100%)
	*ebp*S		23 (100%)	3 (100%)	2 (100%)	0	9 (100%)	7 (100%)	1 (100%)	2 (100%)	0	0	1 (100%)	7 (100%)
	*eno*		23 (100%)	3 (100%)	2 (100%)	0	9 (100%)	7 (100%)	1 (100%)	2 (100%)	0	0	1 (100%)	7 (100%)
	*fib*		23 (100%)	3 (100%)	2 (100%)	0	1 (11.1%)	4 (75.1%)	1 (100%)	2 (100%)	0	0	1 (100%)	7 (100%)
	*fnb*A		23 (100%)	3 (100%)	2 (100%)	0	9 (100%)	7 (100%)	1 (100%)	2 (100%)	0	0	1 (100%)	7 (100%)
	*fnb*B		3 (13.0%)	2 (66.6%)	0	0	4 (44.4%)	3 (42.8%)	1 (100%)	2 (100%)	0	0	1 (100%)	7 (100%)
	*map*		23 (100%)	3 (100%)	2 (100%)	0	9 (100%)	7 (100%)	1 (100%)	2 (100%)	0	0	1 (100%)	7 (100%)
	*sas*G		7 (30.4%)	2 (66.6%)	0	0	5 (55.5%)	6 (85.7%)	1 (100%)	2 (100%)	0	0	1 (100%)	7 (100%)
	*sdr*C		23 (100%)	3 (100%)	2 (100%)	0	9 (100%)	7 (100%)	1 (100%)	2 (100%)	0	0	1 (100%)	7 (100%)
	*sdr*D		15 (65.2%)	2 (66.6%)	2 (100%)	0	7 (77.7%)	6 (85.7%)	1 (100%)	2 (100%)	0	0	1 (100%)	6 (85.7%)
	*vwb*		23 (100%))	3 (100%)	2 (100%)	0	9 (100%)	7 (100%)	1 (100%)	2 (100%)	0	0	1 (100%)	7 (100%)
**Regulatory genes**	*agr*	I	5 (21.7%)	2 (66.6%)	0	0	7 (77.7%)	4 (57.1%)	1 (100%)	0	0	0	1 (100%)	1 (14.2%)
		II	3 (13.0%)	0	0	0	1 (11.1%)	2 (28.5%)	0	1 (50%)	0	0	0	3 (42.9%)
		III	15 (65.2%)	1 (33.3%)	2 (100%)	0	1 (11.1%)	1 (14.2%)	0	1 (50%)	0	0	0	3 (42.9%)
		IV	0	0	0	0	0	0	0	0	0	0	0	0
	*sar*A		23 (100%)	3 (100%)	2 (100%)	0	9 (100%)	7 (100%)	1 (100%)	2 (100%)	0	0	1 (100%)	7 (100%)

**Table 4:** Correlation between slime production ability using CRA assay and distribution of biofilm-associated genes, MSCRAMMs genes, and regulatory genes in *S. aureus* isolates.

Criteria			Sheep Mastitis Isolates (*n* = 28)		Human Isolates (*n* = 19)		Food Isolates (*n* = 8)	
			Slime Producing (18)	No Slime Producing (10)	Slime Producing (13)	No Slime Producing (6)	Slime Producing (8)	No Slime Producing (0)
**Polysaccharide intercellular adhesin and biofilm-associated protein genes**	*ica*ACD		18 (100%)	10 (100%)	13 (100%)	6 (100%)	8 (100%)	0
	*bap*		0	0	0	0	0	0
**Genes encoding microbial surface components recognizing adhesive matrix molecules (MSCRAMMs)**	*bbp*		17 (94.4%)	10 (100%)	12 (92.3%)	6 (100%)	6 (75.0%)	0
	*clf*A/B		18 (100%)	10 (100%)	13 (100%)	6 (100%)	8 (100%)	0
	*cna*		4 (22.2%)	2 (20.0%)	9 (69.2%)	4 (66.6%)	4 (50.0%)	0
	*ebh*		18 (100%)	10 (100%)	9 (69.2%)	3 (50.0%)	8 (100%)	0
	*ebp*S		18 (100%)	10 (100%)	13 (100%)	6 (100%)	8 (100%)	0
	*eno*		18 (100%)	10 (100%)	13 (100%)	6 (100%)	8 (100%)	0
	*fib*		18 (100%)	10 (100%)	6 (46.1%9	2 (33.3%)	8 (100%)	0
	*fnb*A		18 (100%)	10 (100%)	13 (100%)	6 (100%)	8 (100%)	0
	*fnb*B		18 (100%)	2 (20.0%)	8 (61.5%)	2 (33.3%)	8 (100%)	0
	*map*		18 (100%)	10 (100%)	13 (100%)	6 (100%)	8 (100%)	0
	*sas*G		6 (33.3%)	3 (30.0%)	10 (76.9%)	4 (66.6%)	8 (100%)	0
	*sdr*C		18 (100%)	10 (100%)	13 (100%)	6 (100%)	8 (100%)	0
	*sdr*D		13 (72.2%)	6 (60.0%)	12 (92.3%)	4 (66.6%)	7 (87.5%)	0
	*vwb*		18 (100%)	10 (100%)	13 (100%)	6 (100%)	8 (100%)	0
**Regulatory genes**	*agr*	I	5 (27.7%)	2 (20.0%)	7 (53.8%)	5 (83.3%)	2 (25%)	0
		II	1 (5.5%)	2 (20.0%)	3 (23.0%)	1 (16.6%)	3 (37.5%)	0
		III	12 (66.6%)	6 (60%)	3 (23.0%)	0	3 (37.5%)	0
		IV	0	0	0	0	0	0
	*sar*A		18 (100%)	10 (100%)	13 (100%)	6 (100%)	8 (100%)	0
